# A Review of 3D Printing in Dentistry: Technologies, Affecting Factors, and Applications

**DOI:** 10.1155/2021/9950131

**Published:** 2021-07-17

**Authors:** Yueyi Tian, ChunXu Chen, Xiaotong Xu, Jiayin Wang, Xingyu Hou, Kelun Li, Xinyue Lu, HaoYu Shi, Eui-Seok Lee, Heng Bo Jiang

**Affiliations:** ^1^The Conversationalist Club, School of Stomatology, Shandong First Medical University & Shandong Academy of Medical Sciences, Tai'an, Shandong 271016, China; ^2^Department of Oral and Maxillofacial Surgery, Graduate School of Clinical Dentistry, Korea University, Seoul 08308, Republic of Korea

## Abstract

Three-dimensional (3D) printing technologies are advanced manufacturing technologies based on computer-aided design digital models to create personalized 3D objects automatically. They have been widely used in the industry, design, engineering, and manufacturing fields for nearly 30 years. Three-dimensional printing has many advantages in process engineering, with applications in dentistry ranging from the field of prosthodontics, oral and maxillofacial surgery, and oral implantology to orthodontics, endodontics, and periodontology. This review provides a practical and scientific overview of 3D printing technologies. First, it introduces current 3D printing technologies, including powder bed fusion, photopolymerization molding, and fused deposition modeling. Additionally, it introduces various factors affecting 3D printing metrics, such as mechanical properties and accuracy. The final section presents a summary of the clinical applications of 3D printing in dentistry, including manufacturing working models and main applications in the fields of prosthodontics, oral and maxillofacial surgery, and oral implantology. The 3D printing technologies have the advantages of high material utilization and the ability to manufacture a single complex geometry; nevertheless, they have the disadvantages of high cost and time-consuming postprocessing. The development of new materials and technologies will be the future trend of 3D printing in dentistry, and there is no denying that 3D printing will have a bright future.

## 1. Introduction

In 1986, Charles Hull introduced the first three-dimensional (3D) printing technology, and the industry developed many different manufacturing technologies, which have been applied to numerous fields [1–4]. In 1986, Hull patented stereolithography (SLA) and built and developed a 3D printing system. In 1990, Scott Crump received a patent for fused deposition modeling (FDM) [5]. Since then, 3D printing has been increasingly progressing.

Three-dimensional printing, the alias of additive manufacturing [6], is an advanced manufacturing technology. It is based on computer-aided design (CAD) digital models, using standardized materials to create personalized 3D objects through specific automatic processes [2, 7, 8]. It is used for rapid prototyping, which has been widely used in industry, design, engineering, and manufacturing fields for nearly 30 years. With the rapid development of new materials, printing technologies, and machines, 3D printing is likely to completely change the traditional teaching and experimental modes [5].

In the field of medicine, such as traumatology, cardiology, neurosurgery, plastic surgery, and craniomaxillofacial surgery, 3D printing is often used for digital imaging in surgical planning, custom surgical devices, and patient-physician communication [9]. In the field of dentistry, its applications range from prosthodontics, oral and maxillofacial surgery, and oral implantology to orthodontics, endodontics, and periodontology [10, 11].

Compared with traditional wax loss technology and subtraction computer numerical control methods, 3D printing has advantages in process engineering [12]. Owing to its rapid production, high precision, and personal customization, complete dentures, and implant teeth are easier to obtain [13]. Additionally, the applications of 3D printing in dentistry can assist in providing patients with lower cost and more personalized services and simplify the complex workflow related to the production of dental appliances [7]. For example, before the popularization of 3D printing technologies, the restoration was generally fabricated by milling. Currently, 3D-printed restorations have shown several advantages. Some studies have shown that the edge and internal gap values of 3D printing restorations are significantly lower than those of milling restorations [8]. For example, dental crowns are generally fabricated from traditional plaster models, and currently, dental crowns fabricated from 3D printing models are also popular. However, a recent study has shown that the fit of crowns fabricated using 3D printing is lower than that using the plaster model, suggesting that 3D printing technologies are novel technologies with a lack of research; therefore, the processing of 3D printing materials is still controversial [14].

The 3D printing technologies can quickly accept CAD data. Moreover, it can rapidly manufacture single and small-batch parts, new samples, complex shape products, molds, and models [15]. It has many advantages, such as high material utilization, high economic benefits, and the production of certain scale products on demand. However, it still has several disadvantages, such as high cost of processing and material and time-consuming postprocessing. Still, in general, 3D printing has been successfully applied in the medical field [16, 17].

This review discusses the three main 3D printing technologies, including powder bed fusion (PBF), light curing, and fused deposition modeling (FDM). The factors affecting the accuracy of 3D printing including process parameters and material composition will also be discussed. Finally, this review describes the applications in dentistry of 3D printing in detail, including manufacturing working models and primary applications in the fields of prosthodontics, oral and maxillofacial surgery, and oral implantology.

## 2. Three-Dimensional Printing Techniques

According to different working principles, 3D printing technologies can be divided into three categories: PBF, light curing, and FDM. As illustrated in Table 1, they can be refined into specific technologies, each with its distinctive advantages.

### 2.1. Powder Bed Fusion (PBF)

Any powdered material, which can be sintered or fused by laser radiation and solidified by cooling, could be suitable for laser sintering or fusion technologies [18]. According to the energy sources and powder materials, PBF is divided into the following printing technologies: selective laser melting (SLM), selective laser sintering (SLS), electron beam melting (EBM), and direct metal laser sintering (DMLS) [19]. All these technologies use heat to melt powdered materials [20]. In dentistry, PBF is used to manufacture all kinds of metal products including AM titanium (Ti) dental implants, custom subperiosteal Ti implants, custom Ti mesh for bone grafting-techniques, cobalt-chromium (Co-Cr) frames for implant impression procedures, and Co-Cr and Ti frames for dental implant-supported prostheses [20]. Moreover, PBF shows considerable potential for manufacturing ceramic restorations [21], which can be used to manufacture frame crowns, model casting abutments, and models.

The definitions of the terms “laser sintering” and “selective laser melting” are inconsistent [18]. The operating ambient temperatures of SLS and DMLS do not reach the materials' melting points. The metal powder is partially melted, which results in a large porosity and a rough surface [22]. However, in the SLM process, the powder melts directly at the melting point. Another technique, EBM, differs from SLM by using an electron beam to melt the material. Both technologies completely melt the metal powder in an inert build chamber containing purified argon gas [22]. PBF uses the roller to apply the powdered substrate from the reservoir onto the build platform. Thereafter, a laser or electron beam selectively fuses the powder particles according to the cross-sectional configuration of the CAD file being produced. The shapes of the build platform descend by orders of magnitude in the thickness of the printed layers, and then the process goes in cycles until the object has finally been built [21].

Ti and its alloys are particularly suitable for 3D printing technologies, particularly SLS [1]. Studies have demonstrated that Ti structures fabricated using 3D printing technologies have great yield strength, ultimate tensile strength, and excellent ductility [1]. Ceramics can also be used in SLS; however, manufacturing ceramics for dental applications employ an indirect technical measure that relies on polymer bonding to fuse ceramic particles. The molded parts produced are fully cleaned and sintered [21]. SLM does not require any debinding process as it does not involve binders to produce intermediate green fragments. The fabrication time based on PBF is also shorter than that of other 3D printing technologies [19]. However, higher heating and cooling rates may lead to thermal shock and rupture. This can be avoided by preheating the powder. SLS-based products can be weak and porous and require complex postprocessing. A variation based on this technique is known as DMLS whose products are quite dense. Ciocca et al. presented an innovative multidisciplinary approach to the restoration of atrophied maxillary dental arches using DMLS's custom Ti mesh to guide bone regeneration [23].

### 2.2. Light Curing

Light curing technology is a general term for a type of 3D printing technologies using photosensitive resin materials that are cured and molded under light irradiation [24]. It consists of three main technologies: SLA, digital light processing (DLP), and photo jet (PJ). The printing process in SLA and DLP technologies can be divided into three discrete procedures: light exposure, building platform movement, and resin refilling.

**Figure 1:**
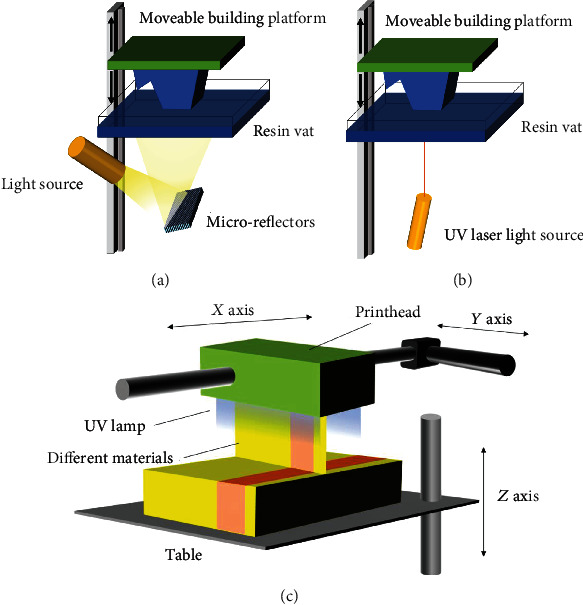
Schematic diagram of three-dimensional (3D) technologies. (a) Digital light processing. (b) Stereolithography. (c) Fused deposition modeling.

SLA is one of the earliest practical 3D printing technologies, and its device consists of a reservoir for the material supplier of photosensitive liquid resin, a model build platform, and an ultraviolet (UV) laser to cure the resin. In the building process, the build platform is submerged in a liquid resin, and the resin is polymerized using a UV laser. Then, the build platform moves a distance equivalent to the thickness of one layer, and the uncured resin then covers the previous layer (Figure 1(a)) [1, 21]. There are two ways to move the platform in SLA technology. The first is the top-down movement of the platform. A layer of resin covers the construction platform that is soaked in the resin reservoir. After scanning the first layer with the laser, the construction platform moves down, and a new layer of resin is added by a wheel next to it. The build cycle is repeated until the object is created. In contrast, in the platform-bottom-up approach, the platform is submerged at the bottom of the resin reservoir, and the gap between the platform and bottom can spread only a single layer of resin. The laser is placed at the bottom of the reservoir, and the resin layer would be scanned. After curing, the platform increases the distance of one layer, and the resin material can completely fill the gap between the platform and the bottom owing to gravity. The platform-bottom-up approach has several advantages over the platform-top-down approach. First, in the second approach, the resin is in direct contact with oxygen as it undergoes polymerization, whereas light-curing occurs at the bottom to avoid oxygen interference in the platform-bottom-up approach. Second, the laser is located at the bottom, which reduces the potential for injury to the operators. Third, the resin can be refilled automatically owing to gravity. Therefore, most SLA printers currently introduce this technology [25].

In the case of ceramics, SLA incorporates ceramic particles into a curing resin that selectively cures a ceramic slurry. As the viscosity of the slurry affects the mechanical properties of the structure, the ratio of the ceramic powder content to the resin needs to be balanced. Ceramics with different chemical compositions, such as alumina and zirconia, have good mechanical resistance and are suitable for polycrystalline ceramic crowns [26]. Therefore, this kind of ceramics is the focus of research and development of SLA.

The DLP technology microsystem consists of a rectangular arrangement of mirrors, called a digital microreflector device. Each mirror represents one pixel, and the resolution of the projected image depends on the number of mirrors. The angles of the microreflectors are adjusted individually. The light emitted by the light source is refracted by the micromirror and then projected onto the surface to be printed as one pixel (Figure 1(b)) [19, 21]. Compared to scanning the layer sequentially using a laser in SLA technology, the advantage of DLP is that the entire layer can be constructed by single laser irradiation. As each layer is constructed independently of the respective layer shape or the number of pixels, the construction time can be reduced.

Unlike the above two patterns of polymerizing liquid monomers and oligomers at specific locations, the principle of PJ is a photopolymerizable inkjet. During the printing process, the printhead moves along the *X*/*Y*-axis, and the photopolymer is sprayed on the table, while an ultraviolet lamp emits light along the direction of movement of the printhead to cure the photopolymer on the building surface to complete one layer of printing. The table then descends one layer along the *Z*-axis, and the device repeats the build cycle until the object is printed (Figure 1(c)). The distinctive feature of this technology is the diversity of materials, from thermoplastics to resins and ceramics, even zirconia paste. All the materials listed can be printed and fused, which is a unique advantage over other technologies. Moreover, inkjet-based 3D printing allows for the blending of materials by printing different materials in the same position, by which it can form objects with a variety of properties [27]. The surface quality and print resolution of the objects manufactured by photopolymer injection technology are particularly high and do not require any small layer thickness for surface polishing.

### 2.3. Fused Deposition Modeling

FDM is one of the most popular and cheap 3D printing technologies in dentistry [28]. The filamentous thermoplastic material is heated and melted by the nozzle. Under the control of the computer, the nozzle and worktable move in the *X*- and *Y*-axis directions, respectively, and the material in the molten state is extruded and finally solidified through the accumulation of materials layer-by-layer to form the product [1, 26, 29].

Polylactic acid (PLA), polycarbonate, and polyamide, acrylonitrile-butadiene-styrene copolymers are some of the engineering thermoplastics commonly used for FDM applications [29]. PLA is more environmentally friendly and suitable in the oral cavity [1]. Yefang et al. mentioned that medical-grade polycarpic acid-tensioned-tricalcium phosphate scaffolds constructed using FDM are biocompatible and have high mechanical strength and can be used as tissue scaffolds in dentistry [30]. Moreover, Chen et al. demonstrated that custom pallets produced by FDM technology can fit plaster models [31].

## 3. Factors Affecting 3D Printing Products

Various factors, such as printer process parameters, material composition, and postprocessing, affect printed products, which mainly manifest in accuracy, processing time, and material properties such as ultimate tensile stress, elasticity modulus, yield strength, impact strength, and induced residual stress.

### 3.1. Process Parameters

Process parameters, including build orientation, layer thickness [32–35], and postcuring [36], affect the printing results.

#### 3.1.1. Build Orientation

The setting of the build orientation affects material properties, product accuracy, and biocompatibility.

For SLA, Quintana et al. explored the impacts of different construction orientations on the ultimate tensile stress as well as the modulus of elasticity in the tension of SLA-fabricated samples. They constructed samples with different axes (parallel to the *X*- or *Y*-axis, or at an angle of 45° to the dual axes); layouts (samples were flat or edge-built) [37]; and locations (differential distances from the center of the platform). The results demonstrate that the ultimate tensile stress and modulus of elasticity are not significantly affected by the axis and position, but the layout settings have a remarkable effect on both properties. Samples built on the edge showed better performance (3.53% vs. 4.59%) compared to the other placement method [37]. Another study found that the printed layer direction being perpendicular to the load direction was better than being parallel in terms of the compressive strength of the material [38], a view also supported by Chockalingam et al. [34].

**Figure 2:**
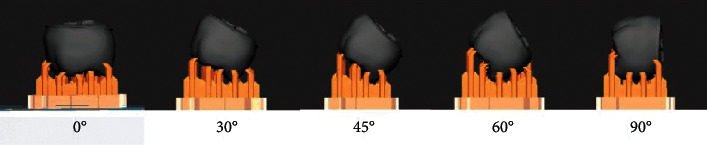
Crowns are made with digital light processing at the setting of five different construction angles to investigate the effect of construction angle on the accuracy of products [32].

For accuracy, Alharbi et al. [38] used SLA technology to fabricate crowns with various constructive angles, and the results showed that an angle of 120° provided greater dimensional accuracy and minimal support surfaces for crowns. However, Osman et al. [39] found that when full-coverage dental restorations were fabricated using DLP, the color map and root-mean-square estimate showed the most favorable deviation pattern and the highest dimensional accuracy at a tectonic angle of 135°. Similarly, Park et al. used DLP technology to print three-unit prostheses using two implants in different construction directions (Figure 2) and observed that when the construction angle was set to 45° or 60° (corresponding to the aforementioned 135° and 120°), the internal gap was smaller and the fit was higher [32].

For FDM, additionally, all planes of samples printed using SLM technology fulfill the biocompatibility requirements, and the roughness amplitude parameters (Ra) of horizontal and diagonal printing are relatively high, which can increase the activity of surface fibroblasts [40]. Moreover, for FLM technology, horizontal printing could fabricate samples with greater hardness [11].

#### 3.1.2. Layer Thickness

The most suitable layer thickness may be different for each printing technologies.

For SLA, when it comes to the influence of layer thickness setting on the samples, Cheng et al. [41] mentioned that the fewer the number of slices (the thicker the layer), the shorter the construction time; however, meanwhile, the lower the accuracy. It has been reported that when using SLA technology to print samples with a decrease in layer thickness, the strength of the sample increases [33]. Another study conducted by Chockalingam et al. [34] indicated that under the settings of 60 min postcuring time and vertical construction, the optimal layer thickness was 100 *μ*m layer thickness. Loflin et al. used the Cast-Radiograph Evaluation grading system, an objective method for the evaluation of treatment outcomes of cases presented for the clinical examination, to evaluate final orthodontic samples with different layer thickness settings; the results demonstrated that a layer thickness of 100 *μ*m was the best choice [42]. For FLM printing, Prechtel et al.'s research indicated that a thickness of 200 *μ*m was the best choice when considering quality and production time [11].

#### 3.1.3. Infill Ratio

The infill ratio is the solid volume fraction of the printed part. The printer provides the ideal infill ratio by adjusting the air gap between the printed lines [43]. Compared with SLS and SLM, the construction of filling rate parameters is critical for FDM technology [44]. Zaman et al. found that for FDM, the filling percentage is the key factor affecting the compressive strength [45]. Ali et al. studied the influence of five different filling rates in the range of 20%–100% on the mechanical properties of FDM printed products. Studies have shown that with the increase in the filling rate, the strength increases. When the filling rate is low, a higher strength value can be achieved through careful design and construction of the structure [43]. Currently, there are many FDM machines available in the market, each with its process parameter settings, which will affect the relevant quality characteristics of the produced parts. Based on the existing literature, to obtain high-quality and enhanced performance, the optimization of FDM process parameters is one of the most critical design tasks [46].

#### 3.1.4. Other Parameters

The balling phenomenon generated by the fragmentation of the remelted line is a major drawback of SLM technology. Therefore, the parameters greatly influence the quality of the product [47]. At a scanning rate of 128.6 mm/s and pulse power of 200 W of the laser, the beads with the optimal profile are produced, and when the scanning line spacing is 100 *μ*m, the 3D Co-Cr body surface is the smoothest. Simultaneously, research by Prechtel et al. showed that FLM exhibited high indentation hardness and modulus at a printing speed of 1200 mm/min [11]

### 3.2. Material Composition

Materials are critical to the performance of the 3D printed process and the products produced. Many researchers have studied the effect of various additive components on material conversion and properties in order to improve printed materials [48].

Vitale et al. found that the addition of the dye affected the reaction kinetics in acrylic resins; that is, with the increase in the dye concentration, the conversion reaction slows down [49], and conversely, 4,4′-bis (N,N-diethylamino) benzophenone could be added to the resin as a coinitiator, which results in a much higher conversion rate at each time point than before. Wang et al. [50] found that surface-modified sepiolite nanofibers, graphene oxide, and TiO₂ and SiO₂ nanoparticles could be used as complementary fillers in epoxy-based resins to enhance the mechanics of the resins, particularly sea foam nanofibers, which can increase the tensile strength of resins by 41.4% and lead to an increase in the hardness of nanocomposites to 112 heat release rate, which is suitable for fabricating high-precision oral models.

**Figure 3:**
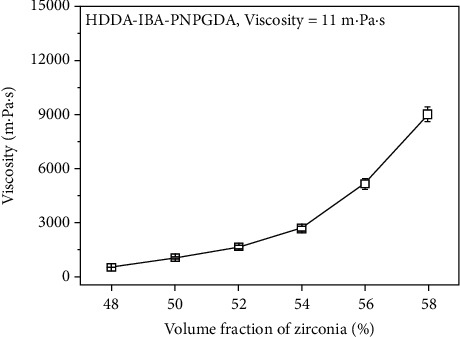
This figure shows the viscosity of zirconia suspension according to volume fraction, which reveals a pattern that with the increase in the volume fraction of zirconia, the viscosity also increases. The highest viscosity of 9025 ± 57 mPa is measured at the highest volume fraction of 58 vol% [56].

Several studies have aimed to improve the performance of scaffold materials. Zhao et al. found that biphasic calcium phosphate ceramic scaffolds showed better compressive strength values, elastic modulus, seeding efficiency, cell proliferation, and differentiation capability than both pure *β*-tricalcium phosphate (*β*-TCP) and hydroxyapatite (HA) scaffolds [51]. An increase in the weight ratio of HA (wt%) reduced scaffold degradation and was optimal for cell proliferation at 40%, whereas at 60%, the scaffold exhibited optimal osteogenic differentiation. Fielding et al. [52] and Ke et al. [53] added silica (SiO₂) and zinc oxide (ZnO) dopants to TCP scaffolds and produced scaffolds with increased density, 2.5-fold higher compressive strength, and better cell proliferation compared to pure scaffolds. Prechtel et al. reported that polyaryletherketone filled with TiO₂ had higher Martens parameters, that is, exhibiting higher hardness [11]. Cheah [54] improved the mechanical strength of prototypes by adding 20% of the concentration of short glass fibers to the polymer material, which was also supported by the study by Karalekas and Antoniou [55]. Jang et al. demonstrated that an increase in the volume fraction of zirconia reduced the depth of cure and the bending strength from 94 MPa at 48 vol% to 674 MPa at 58 vol% (Figure 3) [56]. Ottemer and Colton's study demonstrated that aluminum-filled epoxy resins had almost no reduction in mechanical properties and glass transition temperature in humid environments compared to conventional resins owing to the reduced moisture absorption influence of aging on epoxy-based fast mold materials [57].

### 3.3. Postprocessing

Proper postprocessing can improve the performance of printed samples with higher cost and more time consumption.

The shrinkage and deformation of resin materials have limited the development of SLA; however, the postcuring process evades this disadvantage.

After curing indicates that the cured resin object is exposed to either curing temperature or above for a long time [36], UV and microwave postcuring can improve elastic modulus and ultimate strength of samples. Simultaneously, the growth in laser power can also increase the strength of the sample [54]. Jindal et al. [36] used a 405 nm light source (13 poly-directional light-emitting diodes) to postcure printed clear dental aligners and found that the curing time of 15–20 min at 40–80°C significantly improved the resin's ability to resist pressure loading [36].

**Figure 4:**
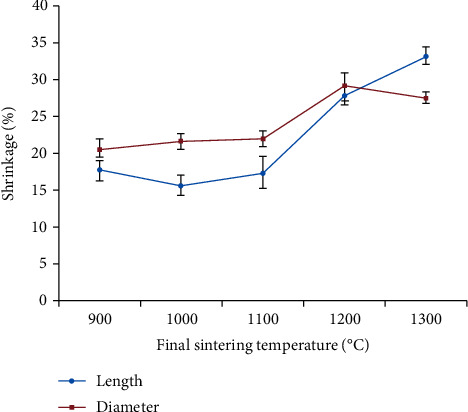
Influence of final sintering temperature on shrinkage of three-dimensional- (3D-) printed titanium samples. The shrinkage of length decreases from 900°C to 1000°C and increases from 1000°C to 1300°C, while the shrinkage of diameter increases from 900°C to 1200°C and decreases from 1200°C to 1300°C, and the range of changes is not equal [58].

For FDM, Wang et al. showed that laser polishing resulted in higher corrosion resistance of the samples, approximately 30% higher than that of the heat-treated samples [50]. Gagg et al. studied the influence of the final sintering temperature on the morphology and mechanical properties of 3D printed samples, using 3D printing and sintering technologies to fabricate porous Ti samples at different temperatures, and found that the shrinkage rate was approximately 20% at the final sintering temperatures below 1100°C; however, it increased dramatically to 20% at 1300°C, and sample hardness and yield strength increased with increase in the final sintering temperature, while the elasticity modulus remained stable (Figure 4) [58].

### 3.4. Aging

Aging refers to the process of a series of changes in the chemical composition and structure of polymer materials due to environmental factors. These changes can cause a transformation in material properties.

For SLA, Ottemer and Colton's study of the effect of aging on light-curing resins showed that the mechanical properties of the resin and glass transition temperature decreased in a humid environment, while aging time had no significant effect on these properties [57]. Another study showed that the elasticity modulus, ultimate tensile stress, flexural modulus, and strength all increased with the extension of aging time; however, the impact strength and elongation at break decreased [59]. A similar experiment investigated the dimensional changes of light-curing resins at different temperatures and humidity levels and showed that slight changes in sample size were insignificant when the relative humidity varied between 20% and 90%, while the growth in ambient temperature increased the moisture absorption capacity of the resin, resulting in significant growth in the sample size [60].

Prechtel et al. subjected samples printed by FLM to thermal cycling between 5 and 55°C and hydrothermal aging for 2 h to simulate in vivo conditions for 15–29 years and found a decrease in Martens parameters, that is, a decrease in hardness [11].

### 3.5. Additional Factors

In addition to some of the above factors, many other factors affect the 3D printed samples.

Di Fiore et al. [61]. compared pre- and postceramic firing edge gaps in 3D-printed Co-Cr frameworks and found that the edge gaps of postceramic firing were larger, however still lower than 120 *μ*m within the clinically acceptable limit.

**Figure 5:**
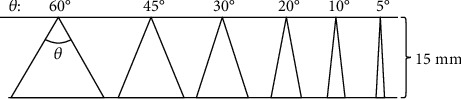
Triangular models with different degree of sharpness (60°, 45°, 30°, 20°, 10°, and 5°). The height of all models is set at 15 mm, and the width of the bottom edge changes with the degree of sharpness [62].

Using a prism model, Ide et al. investigated the accuracy of parts of the model with different degrees of sharpness (60°, 45°, 30°, 20°, 10°, and 5°) and found that the smaller the angle, the less accurately the part can be reproduced (Figure 5) [62]. Torok et al. evaluated the impact of disinfection and sterilization on 3D printed surgical guides, and the results showed that only autoclave sterilization (134°C) significantly increased the stiffness of the samples among the numerous sterilization methods [63].

Zhao et al. examined the impact of macropore percentage (Pmacro) on scaffolds and showed that with the increase in the Pmacro, the scaffolds' degradability increased [51]. Pmacro for 50% of the scaffolds was best for cell proliferation, while 30% of the scaffolds showed the greatest promotion of osteogenic differentiation.

## 4. Clinical Applications

Manufacturing working models for diagnosis and surgical treatment appear to be the most common use of 3D printing technologies. Additionally, as shown in Table 2, 3D printing technologies not only have a variety of clinical applications in prosthodontics, maxillofacial surgery, oral implantology, and other fields but also have great potential with distinctive advantages.

### 4.1. Working Models

In traditional orthodontics, the research model of orthodontic treatment schemes exists in the form of a physical gypsum model, which is easy to lose, break, and degenerate [64]. In digital orthodontics, 3D printing is mainly performed using oral scanners, portable cameras, computers, and orthodontic software, all of which can be used to construct dental arches. The five most popular printing technologies are FDM, PJ, SLA, SLS, and DLP [65, 66].

Compared with plaster models, the working models prepared using 3D printing technologies have many advantages, including the lighter weight, lower damage probability to most of the materials, better durability, higher wear resistance, and the share of digital data. Additionally, environmentally friendly materials can be used for printing [67]. Jeong et al. [68] found that compared with traditional plaster models, 3D printed models have more advantages in accuracy and reproducibility, and compared with milling, 3D printed models have higher accuracy. Moreover, many researchers have conducted extensive research on the accuracy and reproducibility of digital models. They believed that the accuracy of the digital models was slightly lower than that of the original plaster models; however, it was clinically acceptable [69, 70]. Hazeveld et al. [71] found that the assembly of prostheses fabricated on the plaster model was better than that fabricated using 3D printing casting; however, the difference was not significant, and all values were within the range of clinical acceptance. Jang et al. [66] showed that the accuracy of manufacturing fixed teeth on 3D printed models was lower than that of traditional plaster models; however, it was still sufficient for clinical research. The accuracy of the working models varied with the type of 3D printer used. Hazeveld et al. [71] found that DLP was more accurate than diagnostic models prepared using other types of 3D printers.

**Figure 6:**
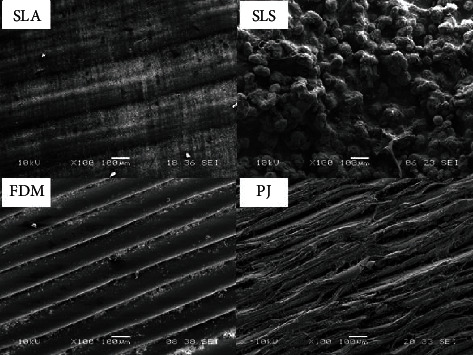
The microscopic images of the different surfaces of three-dimensional (3D) printing technologies. SLA: stereolithography; SLS: selective laser sintering; FDM: fused deposition modeling; PJ: photo jet [65].

Not only metric quality and stability are important attributes of the 3D printed model, but also the surface of the model is also equally significant in prosthodontics, esthetic dentistry, and orthodontics [72]. The FDM model showed clear lines in each layer; however, at higher magnification, some cuts were observed that could affect the durability of the material. The SLS micrograph shows the structures of large and round grains and some holes in the pits. The SLA models had a granular structure; however, their surfaces were homogenous (Figure 6). Jindal et al. [36] found that compared with SLA, FDM provided poor print quality and a layered surface.

**Figure 7:**
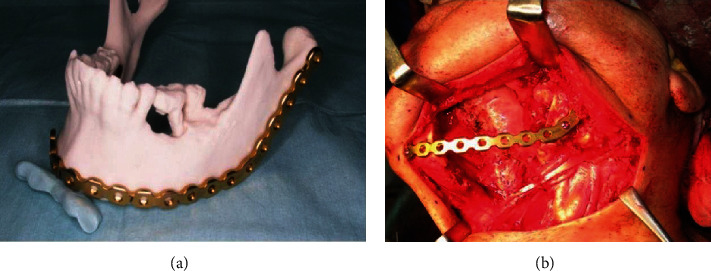
(a) The three-dimensional (3D) medical rapid prototyping model is obtained using powder bed and inkjet head 3D printing with the attached prebent reconstruction plate. (b) The prebent reconstruction plate based on the model is fixed on the residual bone [74].

Moreover, in facial reconstruction, doctors use working models to improve implant fit and design, significantly improving the integrity and esthetics of the reconstructed structure [73]. Azuma et al. found that patients who underwent mandibular reconstruction using biomimetic-based prevent plates had significantly better jaw symmetry than those who underwent conventional reconstructive surgery [74]. Similarly, Paul and D'Urso compared the utility of working models with conventional images in surgery and found that the use of working models significantly increased measurement accuracy, improved surgical planning, and reduced operative time by an average of 17.63% (Figure 7) [75]. Working models also play an excellent role in teaching demonstrations, with 3D printing providing models that have good tolerance, replicability, high fidelity, and patient specificity [76]. Working models can also optimize preoperative surgical planning and reduce surgical procedures; thereby, reducing operative time and risk [77]. Additionally, working models can be used as a reference for intraoperative asepsis and help to interpret volume data, promote team communication, and assist patients in comprehending the operation.

### 4.2. Applications in Prosthodontics

It is undisputed that 3D printing has a great potential for prosthetics, as doctors can prepare, scan, and print their teeth in a session in clinically relevant situations, saving time and money [78]. Using intraoral or extraoral scanners, precise virtual models of the teeth can be prepared. Treatment and restorations can be designed using CAD software. The scanning and CAD design data can be used for milling or printing a variety of dental restorations [79]. This module mainly introduces the applications of 3D printing in prosthodontics, including manufacturing crown and bridge dentures, complete dentures, and removable partial denture frameworks.

#### 4.2.1. Crown and Bridge Dentures

The use of interim crowns is a transitional phase of fixed dentures, which should fulfill mechanical, biological, and esthetic requirements [80]. They have several functions. For example, interim crowns ought to protect periodontal tissues, reduce slight movement of teeth, and maintain occlusal function [81]. The traditional method highly relies on the skills of the operator, and voids created during material mixing can weaken the mechanical strength of manually manufactured temporary crowns, which may eventually lead to fractures [82]. Moreover, there are inherent limitations in machining tools and material properties [83, 84].

**Figure 8:**
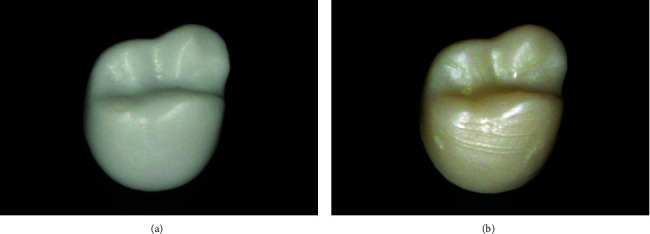
Different surface smoothness of crown restorations reflects the different accuracy of three-dimensional (3D) printing and computer-aided design (CAD)/computer-aided manufacturing (CAM) technologies. (a) The crown restoration is fabricated using 3D printing. (b) The crown restoration is fabricated using subtractive manufacturing [88].

Crown and bridge dentures can be fabricated using resin-based 3D printing technologies such as SLA or DLP [85, 86]. Compared with milling, the amount of materials used in 3D printing technologies is less, with almost no material loss [87]. Furthermore, it can print a variety of materials simultaneously with favorable detail reproducibility [48]. Wang et al. found that the external trueness of 3D printing crowns was not less authentic than the actuality of the corresponding milled crowns (Figure 8) [88].

A good fit is essential to guarantee the mechanical stability, durability, and health of surrounding soft tissues [78]. Insufficient adaptability can lead to dental plaque accumulation, microleaking of adhesive, discoloration of edges, and lack of esthetics, tooth sensitivity, dental caries, and periodontal disease [89]. Temporary crowns fabricated using 3D printing were found to have excellent edges and internal fit, which are more accurate than temporary crowns fabricated applying CAD/computer-aided manufacturing (CAM) or traditional milling methods [4, 90]. Pompa et al. found that the edge and internal fit of the temporary crown fabricated using the molding method differed the most [91], followed by the 3D printing and milling method. Aguirre et al. suggested that this may be due to the characteristics of the materials used in the molding method (volume shrinkage during polymerization) [92]. As it was aggregated into a single piece, the difference was even greater [91, 93]. Similarly, Chaturvedi et al. [79] believed that 3D printing improved the coordination of the proximal end, edge, and interior of the temporary crown, and the effect is obvious in the occlusal area. Alharbi et al. [8] believed that the edge and internal clearance values of 3D printed restorations are significantly lower than those of milled restorations, and the lower marginal and internal fit can be attributed to errors caused by the milling tool tolerances [94, 95].

#### 4.2.2. Complete Dentures

The 3D printing technologies can directly receive CAD data and quickly create a new digital model, which can be applied to the fabrication of a complete denture resin base without the need for molds, cutting tools, or tooling fixtures [15].

In addition to 3D printing technologies, subtraction technology and traditional heat-curing and self-curing technologies can also be used in the fabrication of complete denture bases [96]. The combination of polymethylmethacrylate (PMMA) and compression molding technology has been widely used. However, the volume and linear shrinkage of this technology are both higher compared with 3D printing technology [97]. The 3D printing technology has the advantages of faster production of dentures, and there are fewer stages in the work process, which can reduce the possibility of errors [93]. However, the application of 3D printing in the design and development of complete dentures is still under exploration [98].

Correct tissue adaptation is critical for masticatory, performance retention, and the stability of removable dentures [99]. Tasaka et al. [100] reported that the complete denture base fabricated using photopolymerization spray is more accurate than that produced by conventional thermal polymerization. Yoon et al. [101] compared CAD/CAM complete denture base (5-axis milling or DLP generation) with conventional packaging pressing technology (PAP) to evaluate the adaptability of the tissue surface. The results showed that the adaptability of the DLP denture base was slightly better than that of the milling or PAP denture base in the maxilla. Yoon et al. [99]. found that compared with the complete denture bases fabricated using PAP, DLP denture bases show close adaptability in the pressure-bearing area of maxillary arches. DLP and denture bases both show intimate adaptation to the lingual slope of the mandible.

Owing to the possibility of damage to the denture base in real life due to various reasons, high bending strength is critical for the denture base [102]. Aguirre et al. [92] indicated that the flexural strength values of CAD/CAM materials are higher than those of compression-molded denture base materials. In contrast, Ayman [103] and Pacquet et al. [104] found that thermal PMMA had a higher bending strength than CAD/CAM milling denture base material. Additionally, manufacturing methods also affect performance of the denture base. The mechanical properties of 3D printing materials manufactured using denture bases are inferior to those of most milling denture base materials and thermally polymerized acrylic resin [102].

#### 4.2.3. Removable Partial Denture Frameworks

**Figure 9:**
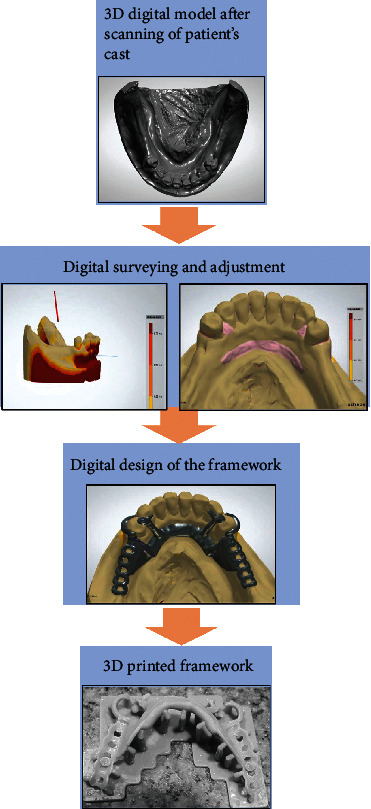
The computer-aided design (CAD)/computer-aided manufacturing (CAM) process of constructing a partial prosthesis framework. (Images from the study of Harb et al. [108])

Rapid advances in CAD/CAM have opened up new avenues of additive and subtractive processes for the fabrication of removable partial denture (RPD) frames [105]. The process of constructing a partial prosthesis frame using CAD/CAM is as follows. First, either an intraoral or extraoral scanner is used to scan impressions or conventional casts to acquire a digital work file such as a standard tessellation file (STL). Second, STL files are transferred to CAD software for designing and finally to 3D printers to generate customized structures (Figure 9) [106, 107].

These new digital workflows have more advantages than traditional workflows [9]. The traditional process of waxing and investing causes wax pattern and refractory cast distortions possibly leading to poor fit of castings compared with new digital workflows [106]. Most importantly, pressure-induced mucosal lesions and residual ridge resorption are the main sources of clinical complications [106]. The use of 3D printing technologies to manufacture RPD enables the denture base to provide more uniform contact pressure and then reducing the risk of long-term bone resorption. Recently, SLM has been shown to produce clinically acceptable RPD frameworks [109]. Tregerman et al. [107] found that when SLM Co-Cr alloy frames were compared with cast or milled RPD frames, the former is regarded as having improved organization and mechanical properties.

### 4.3. Applications in Oral and Maxillofacial Surgery

Combined with advancements in 3D scanning technologies, such as intraoral and extraoral scanning, cone beam-computed tomography (CBCT), and other CAD/CAM technologies, 3D printing has developed rapidly in the field of maxillofacial surgery [110–112]. Three-dimensional printing surgery technologies have many unique advantages, particularly in improving the symmetry and functional effects of craniomaxillofacial plastic surgery techniques [113]. Jacobs and Lin have thoroughly summarized the applications in the craniomaxillofacial region, including surgical guides, occlusal splints, and implants [114].

#### 4.3.1. Occlusal Splints

The occlusal splint is an intraoral device with reversible therapeutic properties. It mainly treats temporomandibular joint disorders by adjusting the occlusal relationship between the upper and lower dental arches [115–117].

The traditional process flow for the production of occlusal splints includes interocclusal wax occlusal registration for the upper and lower dentition of the working models and alginate impressions. This method is costly [117], and errors may occur in the impression process or casting manufacturing process [116]. Manufacturing the occlusal splints by milling not only is time-consuming but also wastes a lot of materials. The shape of the occlusal splints prevents them from nesting well in a round resin blank. One blank can only mill up to two occlusal splints, which causes considerable waste [117, 118]. Additionally, this method has serious wear on the milling tools, particularly hard materials. However, in 3D printing, only the supporting structure must be removed when the occlusal splint is manufactured. Moreover, several splints can be manufactured simultaneously, which greatly improves the manufacturing efficiency and saves time and cost [116]. However, the antistress and antiaging abilities of 3D printing materials are not as good as those of traditional or milling resin materials [116, 118, 119], which will adversely affect their long-term use. Lutz et al. [118] artificially aged 3D printed, milled, or traditionally made occlusal splints in a chewing simulator and found that the 3D-printed occlusal splints had lower wear and flexural resistance than the other two approaches. The 3D-printed occlusal splint material can be used clinically for 1 month, which is consistent with the current approval period for the material [118]. However, for accuracy, milling is similar to 3D-printed occlusal splints [120]. Differences in technologies and materials will affect the performance of the occlusal splints.

#### 4.3.2. Surgical Implants

Among various 3D printing technologies, laser sintering and direct beam melting have become the leading edge in the manufacture of customized porous implants such as customized Ti mesh and reconstruction [23, 71]. The SLS technology has successfully developed a completely biodegradable and bone conductive nanocomposite scaffold with adjustable porosity and mechanical properties [121]. Farré-Guasch et al. [121] have shown that implants manufactured using laser sintering technology can induce the formation of the mandible. Furthermore, Saijo et al. [122] prepared a new customized bone from *α*-tricalcium phosphate powder using an inkjet printer and implanted it in patients with facial deformities.

### 4.4. Applications in Oral Implantology

The application of 3D printing technologies in oral implantology is to develop oral implantology from traditional pure experience modes to digital and accurate modes. They can optimize and simplify the medical treatment process, greatly reduce the technical difficulty and technical risk, and improve the efficiency of dentists.

This module mainly introduces applications in oral implantology, including manufacturing surgical guides and 3D printing custom trays.

#### 4.4.1. Surgical Guides

**Figure 10:**
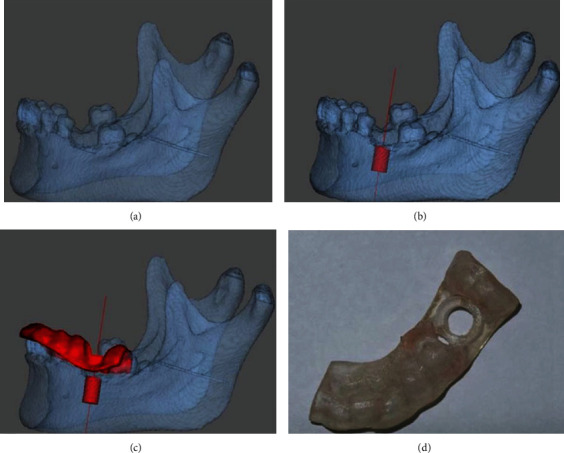
The design and production process of the surgical guide. (a) Digital model of the mandible obtained by scanning. (b) Specify the implant position in the design software. (c) Design surgical guide. (d) Print surgical guide using stereolithography (SLA) [133].

Surgical guides can significantly improve the accuracy and time efficiency in clinical treatment, reduce operation errors, make the treatment results more predictable for dentists, and allow patients to better understand the implant prosthodontic treatment [123]. The surgical guidance systems include dynamic and static systems. Dynamic guides use mechanical or optical systems to transfer virtual plans to the surgical area and display the process on screen in real time [124]. Static surgery guides are fabricated in the laboratory by 3D printing, called SLA guides made by perforations on the jaw models. The static surgery guide differs from the dynamic surgical guide as the former does not move during the operation [125, 126]. The process of making a static surgical guide has changed dramatically. Traditional surgical guides are based on panoramic radiation images. However, the enlargement, distortion, and lack of clarity of the radiation image restrict the creation of the surgical guides, which leads to the inaccuracy and unreliability of the preoperative plan [127]. However, the new surgical guide combined with CBCT, intraoral scanning technology, CAD, and virtual planning environment can be created by combining the digital files obtained [128]. Once the treatment planning is completed by using the application preoperative planning software, the surgical guides can be produced by SLA. The drill guides indicate the insertion position, angles, and depth of the implant, which accurately transfers it to the patient through the simulated plan, establishing a link between the planned and the actual operation while in use (Figure 10) [126, 127]. Optimal dental implant placement can simplify the process of denture restorations, achieve good esthetic effects, and stabilize the hard and soft tissues around the dentures for a long time [127]. Nevertheless, improper dental implant placement can significantly reduce the success rate and long-term predictability of the prosthesis [126]. Therefore, the accuracy of the surgical guides is particularly important. Hermann [129] showed that the correlation error of static-guided surgery was less than that of real-time navigation. However, Jung et al. [130] found no statistically significant differences between them. Tahmaseb et al. [131] compared the accuracy of SLA guides with that of conventional surgical guides in vitro. The average deviation at the entrance of traditional surgical guides is 1.5 mm, the average distance deviation at the vertex is 2.1 mm, and the average distance deviation of SLA is 0.9 mm and 1.0 mm. The results showed that the accuracy of the SLA guide was higher. Al-Harbi and Sun [132] reported that when implants were placed in patients using stereolithographic guides, the implants planned and the actual placement of implants were almost exactly matched in location and axis.

Owing to the advantages of simpler operation and lower investment cost, static techniques are widely adopted as the preferred method to guide the surgery [134]. Currently, most of the major implant brands are based on the same basic principles and have their SLA-guided surgery system. Therefore, compared with a dynamic system, a static system is more frequently used. Briefly, compared with dynamic guides or traditional guides, the SLA guidelines have significant advantages.

#### 4.4.2. Three-Dimensional Printed Custom Trays

**Figure 11:**
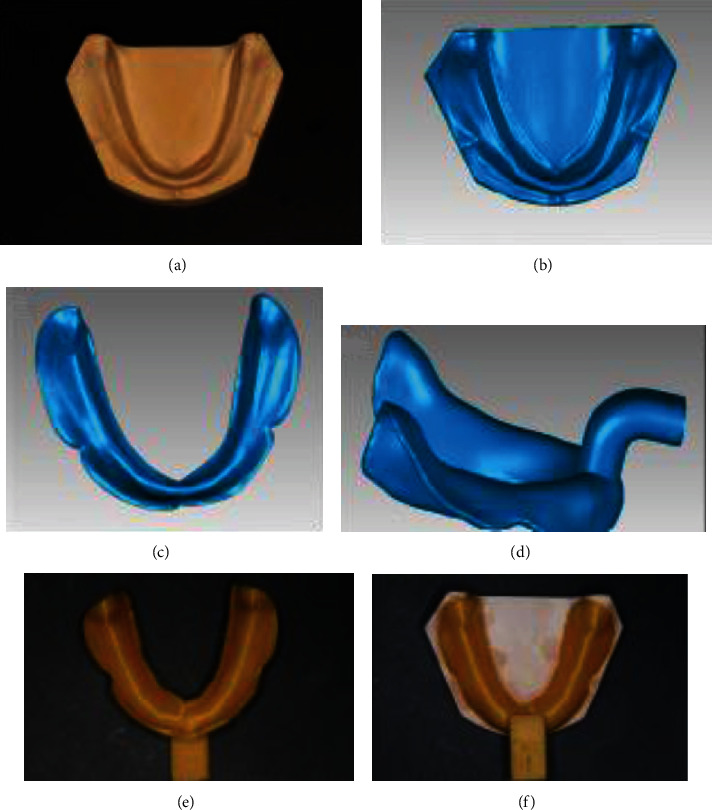
Digital design process and construction of mandibular custom trays. (a) The standard mandibular edentulous cast. (b) Three-dimensional (3D) digital model after scanning of the cast. (c) Trim the edge area of the model. (d) Add handles to the model to form a tray. (e) The custom tray is printed using fused deposition modeling. (f) The tray and the original cast are highly compatible [31].

It is critical to obtain accurate implant impressions for manufacturing prostheses, and accurate edentulous impressions are the basis to ensure that the restoration has good support, retention, stability, restored function, and the ability to protect oral tissue health. The stable position of the trays in the mouth is one of the necessary factors for making accurate impressions. Accurate and stable trays can provide uniform thickness and sufficient space for imprinting materials [135]. Owing to their high precision, shorter processing time [136], and simple program and personalization, 3D printing can produce custom trays [137], which are becoming increasingly popular [138]. With CAD programs and 3D printers, almost all printing work in the production process of custom trays can be completed by the equipment (Figure 11). Three-dimensional printers can also perform multiple tasks to improve their efficiency. Therefore, 3D printing technology can simplify the workflow of direct implant-supported dentures for completely edentulous patients.

Custom trays must meet many requirements. First, the custom trays should have adequate strength [139]. Second, there should be sufficient bonding strength between the tray and the imprinting material [140]. Third, sufficient rigidity must be present to ensure that the impression materials are supported while preventing deformation during casting [141]. Fourth, the printing process should be timely and, finally, sufficiently accurate to ensure accurate impressions [31].

The accuracy of custom trays is an important indicator. When using the custom trays of 3D printing, the space reserved for the impression materials is well controlled with slight deviation, thus improving the accuracy and reproducibility [31]. As shown in the test evaluation, custom trays provide a great improvement in accuracy with an adequate range of extensions and a more uniform 3D space of the impression material compared to the traditional hand-made custom trays [31].

As 3D printing custom trays have several advantages, such as personalization and high precision, they demonstrate greater clinician satisfaction than traditional custom trays. Thus, it is widely used in manufacturing custom implant trays [142]. In the future, 3D printing custom trays can be further promoted in clinical use.

## 5. Conclusion

The appearance and disruptive development of 3D printing technologies bring favorable circumstances to the manufacturing of complex equipment in all walks of life. In the field of dentistry, 3D printing has a wide range of applications, making it possible to create new and more efficient methods for manufacturing dental products. The most common application is to create working models for diagnosis and surgery, followed by a variety of implantable devices, which can help dentists provide patients with more predictable, less invasive, and less costly procedures. For products with complex structures, fine structures, and inconveniences to use mechanical processing technology, 3D printing can use an increasing number of material types and rely on digital data to create complex geometric shapes and accurately fulfill the complex and personalized needs in the dental field. The application of 3D printing technology and CAD software based on 3D imaging and modeling can produce complex geometric shapes and has the advantage of high material utilization.

This review is aimed at providing a practical and scientific overview of 3D printing technologies. In this review, we summarized the classification and characteristics of 3D printing technologies used in dentistry. It also introduced various factors affecting 3D printing based on the first part of the 3D printing technology. It mainly introduced four parts: process parameters, material composition, postprocessing, and aging. The final section presented a summary of the clinical applications of 3D printing in dentistry, including working models and specific applications in prosthodontics, oral and maxillofacial surgery, and oral implantology.

Three-dimensional printing transforms 3D manufacturing into simple 2D superposition, which greatly reduces the complexity of design and manufacturing; however, simultaneously, there will be many defects that affect the performance of products. First, the layer-by-layer superposition principle leads to anisotropy, which leads to different mechanical properties in different directions and restricts the long-term use of intraoral instruments such as occlusal splints. Second, the existence of layer thickness affects the consistency from the digital model to the entity, which makes the surface of the equipment with high surface smoothness requirements, such as ceramic restoration, nonideal. Future research should focus on reducing the negative impact of the 3D printing principle. It is also important to note that 3D printing requires a combination of digital file acquisition equipment and CAD software. The high cost of the equipment makes the popularization of 3D printing a challenge. In contrast, although current 3D printers can print out models in a relatively short period, acquiring digital files takes longer; therefore, 3D printing is not currently applicable to emergency cases. In addition, the accuracy of the printed models is somewhat reduced compared to that of digital files. Additionally, there are some challenges, such as high process, material cost, and time-consuming postprocessing. The lack of well-trained operators may also hinder the application of 3D printing in medical treatment. Moreover, 3D printing technology is suitable for many fields, and most of the machines currently used are not customized for dentistry, which causes some functions to be unsuitable to medical staff. Owing to these drawbacks, 3D printing is still at a competitive disadvantage compared to traditional methods for manufacturing products in bulk. Therefore, 3D printing technology in dentistry should aim to reduce the cost and production time, optimize the surface quality, and improve the process reliability and performance gradient in materials. We anticipate that the use of 3D printers in dentistry will become more specialized and sophisticated in the future.

In the future, new materials and technologies that fulfill dental requirements should be further developed and applied. For example, the Co-Cr alloy material used in the restoration is one of the application materials for DMLS; however, its properties should be further studied to ensure the safety and applicability of the restoration [143]. In addition, in clinical applications, 3D scanners, CBCT, and CT will be better integrated with 3D printing technologies based on their advantages, which will further promote the development of the digital process, not only simplifying the traditional modeling and production process, but can also make the products more accurate, streamlining the production process and lowering the labor cost [128]. In recent years, 3D printing has progressed toward the cellular level, and 3D bioprinting provides unlimited possibilities for the creation of various tissues. The application of 3D printing in oral soft tissue biomaterials has been reflected from experimental to clinical [144]. For example, Nesic et al. described the potential of stem cells, 3D bioprinting, gene therapy, and layered bionic technology, which can be used to regenerate periodontal tissue [145]. To improve the whole CAD/CAM process, machine learning (ML) has been applied to all aspects of the technology [146]. The application of ML algorithms covers all the main aspects that directly affect the quality of the final 3D printed parts, including 3D printing design and other aspects related to the efficiency of the design and manufacturing process [147]. In the near future, ML will be more widely used in the field of 3D printing. Additionally, virtual reality design can interact with 3D printing technologies in the field of dentistry. For example, individuals can directly perform the 3D design of the restoration in the virtual world and observe the 3D restoration products to better estimate the feasibility of the products and reduce the wastage of time and resources. In summary, we anticipate that 3D printing technology will have a bright future.

## Figures and Tables

**Table 1:** Materials and main advantages of 3D printing technologies. The table shows three kinds of 3D printing technologies and their respective classification, materials, and main advantages.

Techniques	Classification	Materials	Advantages
Powder bed fusion	SLM		Require no debinding process [1]
	SLS	Metal materials [2]	Suitable for metal [3]
	EBM		
	DMLS		Produce dense end-products [4]

Light curing	SLA		Good mechanical resistance [5]
	DLP	Resin [6], ceramic [7]	Reduced construction time [5]
	PJ		Higher surface quality of manufactured objects [7]

Fused deposition modeling	Thermoplastic materials [3]		Biocompatible and high mechanical strength of fabricated scaffolds [8]

**Table 2:** 3D printing clinical application classification and main advantages. The table shows the clinical applications and their main advantages of 3D printing technologies in the field of prosthodontics, oral and maxillofacial surgery, oral implantology, and orthodontics and main advantages of each application.

Field	Application	Advantage
Prosthodontics	Crown and bridge dentures	Reducing time consumption [9, 10]
		Good fit [11]
		Good detail reproducible [12]
		Low costs [11]
	Complete dentures	Convenient and fast [13]
		Accurate [14]
		Close adaptability [10]
	Removable partial denture frameworks	Uniform contact pressure [15]
		Good mechanical properties [16]

Oral and maxillofacial surgery	Occlusal splints	Saving time and cost [17]
	Surgical implants	High mechanical strength [18]
		Adjustable porosity [19]
	Prostheses	Convenient and fast [20]
		Accurate [21]
	Working models	Improving the integrity and aesthetics [22]
		Reducing operative time and risk [23]

Oral implantology	Surgical guides	Reducing operation errors [24]
		Simple operation [25]
	Custom trays	High efficiency [26]
		High accuracy [26]

Orthodontics	Working models	Good surface quality [27]
		Light [28]
		High wear resistance [29]

## Data Availability

All data, figures, and tables in this review paper are labeled with references.
